# Association between echocardiographic features and inflammatory biomarkers with clinical outcomes in COVID-19 patients in Saudi Arabia

**DOI:** 10.3389/fcvm.2023.1134601

**Published:** 2023-05-26

**Authors:** Samah I. Abohamr, Rami M. Abazid, Mohammed K. Alhumaid, Abubaker E. Abdulrahim, Mubarak A. Aldossari, Lamiaa Khedr, Rehab H. Werida, Haifa S. Alkheledan, Yazeed S. Aleid, Sara W. Abdelhamid, Abdulmohsen Al Mefarrej, Ahmed W. Abdelhamid, Mohammad Hasan Alaboud, Omar T. Alhasan, Hanem M. Gomaa, Eman Elsheikh

**Affiliations:** ^1^ Department of Cardiology, College of Medicine, Tanta University, Tanta, Egypt; ^2^ Heart Health Center, King Saud medical city, Riyadh, Saudi Arabia; ^3^ Division of Cardiology, Department of Medicine, London Health Sciences Centre, Western University London, Ontario, ON, Canada; ^4^ King Fahad Medical City, Riyadh, Saudi Arabia; ^5^ Clinical Pharmacy & Pharmacy Practice Department, Faculty of Pharmacy, Damanhour University, Damanhour, Egypt; ^6^ Infection Control Administration, King Saud Medical City, Riyadh, Saudi Arabia; ^7^ College of Medicine, Alfaisal University, Riyadh, Saudi Arabia; ^8^Internal Medicine Department, King Faisal University, Alahsa, Saudi Arabia

**Keywords:** echocardiography, COVID-19, outcome, biomarker, thrombus, mortality, VEF, pulmonary hypertension

## Abstract

**Background:**

Respiratory infections are one of the most common comorbidities identified in hospitalized patients. The coronavirus disease 2019 (COVID-19) pandemic greatly impacted healthcare systems, including acute cardiac services.

**Aim:**

This study aimed to describe the echocardiographic findings of patients with COVID-19 infections and their correlations with inflammatory biomarkers, disease severity, and clinical outcomes.

**Methods:**

This observational study was conducted between June 2021 and July 2022. The analysis included all patients diagnosed with COVID-19 who had transthoracic echocardiographic (TTE) scans within 72 h of admission.

**Results:**

The enrolled patients had a mean age of 55.6 ± 14.7 years, and 66.1% were male. Of the 490 enrolled patients, 203 (41.4%) were admitted to the intensive care unit (ICU). Pre-ICU TTE findings showed significantly higher incidence right ventricular dysfunction (28 [13.8%] vs. 23 [8.0%]; *P* = 0.04) and left ventricular (LV) regional wall motion abnormalities (55 [27.1%] vs. 29 [10.1%]; *p* < 0.001) in ICU patients compared to non-ICU patients. In-hospital mortality was 11 (2.2%), all deaths of ICU patients. The most sensitive predictors of ICU admission (*p *< 0.05): cardiac troponin I level (area under the curve [AUC] = 0.733), followed by hs-CRP (AUC = 0.620), creatine kinase-MB (AUC = 0.617), D-dimer (AUC = 0.599), and lactate dehydrogenase (AUC = 0.567). Binary logistic regression showed that reduced LV ejection fraction (LVEF), elevated pulmonary artery systolic pressure, and dilated right ventricle were echocardiographic predictors of poor outcomes (*p* < 0.05).

**Conclusion:**

Echocardiography is a valuable tool in assessing admitted patients with COVID-19. Lower LVEF, pulmonary hypertension, higher D-dimer, C-reactive protein, and B-type natriuretic peptide levels were predictors of poor outcomes.

## Introduction

The coronavirus disease 2019 (COVID-19) pandemic of 2021–2022 has resulted in >664 million confirmed cases and >6 million deaths globally by January 2023 (1). While the respiratory system has been the most directly affected, there is growing evidence that COVID-related heart disease is critical in disease severity and clinical outcomes (2).

The heart muscle is damaged by the severe acute respiratory syndrome coronavirus 2 (SARS-CoV-2) virus and its immunopathological cardiac inflammation consequences (3). It is associated with several direct or indirect cardiovascular complications, such as myocardial damage, myocarditis, heart failure (HF), arrhythmia, and venous thromboembolism (4–6). Myocardial ischemia and necrosis are associated with impaired ventricular function, increasing the risk of mortality in these patients (7). The cause of cardiac involvement may include endothelial dysfunction, cytokine-mediated systemic damage, or stress-induced cardiomyopathy (8, 9). Poor outcomes are associated with higher mortality and cardiac involvement, as indicated by increased troponin T and brain-type-natriuretic peptide (BNP) levels or decreased left ventricular (LV) ejection fraction (EF) (5, 6, 10). Elevated serum troponin levels are present in 17%–36% of COVID-19 patients, indicating myocardial damage (11).

Transthoracic echocardiography (TTE) is a common, low-cost technology for assessing heart anatomy and function. A targeted evaluation provides essential information that can influence clinical decisions in critically ill patients (12, 13).

TTE may provide clues for diagnosing other clinical conditions, such as acute respiratory distress syndrome (ARDS), pulmonary embolism (PE), cardiogenic/non-cardiogenic shock, myocardial infarction, and myocarditis (14–16). Therefore, it is necessary to describe the echocardiographic features of COVID-19 patients and its relationship with biomarkers and poor outcomes. This study aimed to evaluate the possible correlations of echocardiographic findings and inflammatory biomarkers with disease outcomes in hospital-admitted patients with confirmed COVID-19 infections.

## Methods

### Study population

We conducted an observational study that included 490 hospitalized patients. It included all patients aged ≥18 years admitted with confirmed COVID-19 infections who underwent echocardiography at admission between June 2021 and July 2022. Patients aged <18 years, who were COVID-19 negative, patients who have not done echocardiogram and those with suboptimal quality echocardiogram were excluded. All patients were recruited at the Heart Health Center, King Saud Medical City, Riyadh, Kingdom of Saudi Arabia (KSA).

### TTE acquisition

TTE orders from the in-patient service with specific clinical indications were subjected to an additional screening process by cardiologists to assess whether appropriate echocardiographic findings affected the management plan. As recommended by the Saudi Arabian Society of Echocardiography for COVID-19 (17), we followed a simplified protocol that enabled rapid bedside TTE assessments to reduce exposure time (17).

TTEs were performed using a Vivid S70 or Vivid q ultrasound system with an M5 or M5-S phased array probe (GE Healthcare Vingmed Ultrasound AS, Horten, Norway).

LV dimensions, volumes, EF (biplane LV planimetry by the modified Simpson's rule), and mass were assessed. According to the Guidelines and Standards for Cardiac Chamber Quantification by Echocardiography in Adults recommendations, an LVEF of <52% for men and <54% for women suggested abnormal LV systolic function (18).

LV diastolic function was also assessed, according to Nagueh et al. (19), by left atrial volume index (LAVI) and tricuspid regurgitation velocity (TRV), the average ratio of early diastolic mitral inflow velocity to early diastolic mitral annulus velocity (E/e'), and the average ratio between E-wave and A-wave (E/A). In patients with depressed EFs and in patients with normal EFs and myocardial disease, if E/A ratio is <0.8 along with a peak E velocity of <50 cm/sec, then mean left atrial pressure (LAP) is either normal or low and patient has grade I diastolic dysfunction.

If E/A ratio is > 2, LA mean pressure is elevated, grade III diastolic dysfunction is present. Deceleration time (DT) is usually short in patients with heart failure with reduced ejection fraction (HFrEF) and restrictive filling pattern (<160 msec). However, in patients with heart failure with preserved ejection fraction (HFpEF), DT can be normal despite elevated LV filling pressures. If E/A ratio <0.8 along with a peak E velocity of >50 cm/sec, or an E/A ratio > 0.8 but < 2, additional parameters are needed. These include peak TR velocity, E/è ratio and LA maximum volume index. Their cutoff values to conclude elevated LAP are peak velocity of TR jet >2.8 m/sec, average E/è ratio > 14, and LA maximum volume index > 34 ml/m^2^. If more than half or all of the variables meet the cutoff values, then LAP is elevated, and grade II diastolic dysfunction is present. If only one of three available variables meet the cutoff value, then LAP is normal and grade I diastolic dysfunction is present. In case of 50% discordance or with only one available variable, findings are inconclusive to estimate LAP.

In patients with depressed LVEF, pulmonary vein systolic/diastolic (S/D) ratio may be used if one of the three main parameters are not available. A ratio < 1 is consistent with increased LAP.

Right ventricular (RV) systolic function was evaluated using a tricuspid annular plane systolic excursion (TAPSE) of <17 mm, pulsed tissue Doppler S wave of <9.5 cm/sec and RV fractional area change (RV-FAC) <35% (18). Pulmonary hypertension (PH) was diagnosed by using the peak tricuspid regurgitation velocity (TRV) as the key variable for assigning the echocardiographic probability of PH. A peak TRV equal to 2.8 m/s may suggest PH. Then other echocardiographic parameters suggesting PH (including pulmonary flow acceleration time) must be used to assign the probability of PH.

If the TRV is >3.4 m/s then the echocardiographic probability of PH is high. If the TRV is ≤3.4 m/s, then other echocardiographic parameters suggesting PH must be used to assign the probability of PH. These parameters are split into three categories (A: the ventricles; B: the pulmonary artery; C: the inferior vena cava (IVC) and right atrium). Parameters from at least two different categories are needed to determine the probability of PH (Supplementary Material Table S1 and Figure S1). Following the guideline protocol from the British Society of Echocardiography 2018 (20) and the 2022 ESC/ERS Guidelines for the diagnosis and treatment of pulmonary hypertension (21).

All TTE scans were reviewed manually by an expert echocardiographer unaware of the patient's clinical history.

## Specimen collection and serum biomarker measurements

Venous blood samples were collected in serum vacutainer test tubes from each patient between 8 and 9 am after a 30 min rest in the supine position. Blood samples were allowed to clot for 15–30 min and then centrifuged at 3000 rpm for 15 min using a Hettich Zentrifugen EBA 20 centrifuge (Merck, Germany). Blood glucose was measured using the glucose oxidase method. The remaining serum sample was stored at 8°C until measurement of D-dimer, high sensitive C-reactive protein (hs-CRP), BNP, cardiac troponin I, creatine kinase, creatine kinase-myocardial band (MB), and lactate dehydrogenase using commercially available enzyme-linked immunosorbent assays (Sunred Biological Technology, Shanghai, China) according to manufacturer`s instructions.

### Clinical outcomes data collection

The patients' medical history, comorbidities, laboratory data, treatments, and outcomes were extracted from the hospital's electronic medical records. Patients with in-hospital poor outcomes, including PE, HF, ARDS, septic shock, respiratory failure (RF), myocarditis, acute kidney injury (AKI) and death, were assessed. The study evaluating the association between echo-cardiographic features and inflammatory biomarkers with ICU admission and poor outcomes in the enrolled COVID-19 Patients.

### Ethics approval

This study was conducted according to the World Medical Association Declaration of Helsinki and received ethics approval from the Institutional Review Boards of the US Department of Health and Human Services (IORG: IORG0010374) and the King Abdulaziz City for Science and Technology, KSA (registration number: H-01-R-053). The study protocols were approved by the Saudi Arabian Society of Echocardiography for COVID-19 (17).

### Statistical analysis

The data were statistically analyzed using Microsoft Excel 2016 (Seattle, WA, USA) and SPSS Statistics for Windows (version 26.0; IBM Corp., Armonk, NY, USA). The normality of each variable's data was checked using Kolmogorov–Smirnov and Shapiro–Wilk tests. Continuous, normally distributed variables are expressed as Means ± Standard Deviations, and 95% confidence intervals (CIs) and were compared between groups using the Student's *t*-test. Non-normally distributed variables are expressed as median (25th and 75th percentiles) and were compared using the Mann-Whitney U test. Whereas, the categorical variables are expressed as frequency (percent) and were compared using the Chi-squared test. The significance level was set at a *p*-value of 0.05. Predictors of poor outcomes (defined as number of patients who have one or more complications including PE, HF, ARDS, septic shock, RF, myocarditis, AKI and death) were assessed using binary logistic regression analysis. Receiver operator characteristic (ROC) curves were used to assess the inflammatory biomarkers and cardiac enzymes for predicting of Intensive Care Unit (ICU) admission.

## Results

### Baseline characteristics

**Figure 1:**
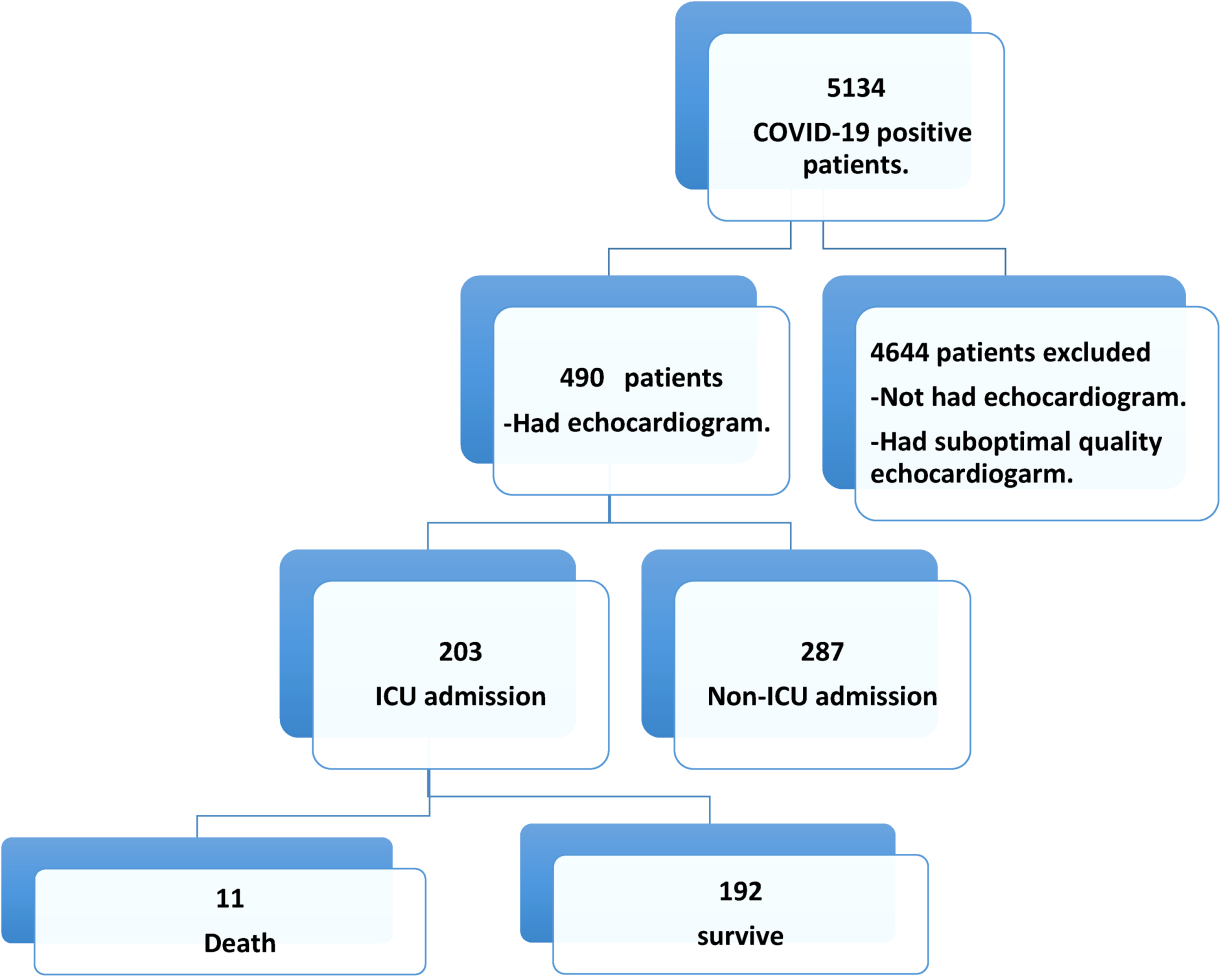
Patients screening and enrollment flow chart.

Over 12 months, 5134 patients were confirmed as COVID-19 positive, of which 490 (9.54%) had TTE scans (Figure 1). Their mean age was 55.6 ± 14.7 years, and 66.1% were male. Nearly half of the patients had diabetes (49.2%) and/or hypertension (52.2%). Their further clinical features are presented in Table 1.

**Table 1:** Patients’ demographics and clinical data outcomes with the risk assessment for ICU admission.

Demographic data:			Descriptive			Risk assessment
		Total *N* = 490	Non-ICU *N* = 287	ICU *N* = 203	*P* value	OR (95% C.I)
^‡^Age		55.6 ± 14.7	55.62 ± 15.22	55.64 ± 13.82	0.9	1.00 (0.99- 1.01)
Sex	Female	166 (33.9)	106 (36.9)	60 (29.6)	0.054	0.72 (0.49–1.05)
	Male	324 (66.1)	181 (63.1)	143 (70.4)		
**History of comorbidities:**						
Smoker		101 (20.6)	52 (18.1)	49 (24.1)	0.11	1.44 (0.93–2.23)
Obesity		68 (13.9)	31 (10.8)	37 (18.2)	0.02*	1.84 (1.10–3.08)
Hypertension		256 (52.2)	146 (50.9)	110 (54.2)	0.47	1.14 (0.80–1.64)
Chronic Kidney Disease		85 (17.3)	45 (15.7)	40 (19.7)	0.25	1.32 (0.83–2.11)
Diabetes Mellitus		241 (49.2)	130 (45.3)	111 (54.7)	0.04**	1.46 (1.02–2.09)
Cerebro Vascular Accidents		74 (15.1)	47 (16.4)	27 (13.3)	0.35	0.78 (0.47–1.31)
Ischemic Heart Disease		63 (12.9)	24 (8.4)	39 (19.2)	< 0.001**	2.61 (1.51–4.49)
Lung Disease		82 (16.7)	32 (11.1)	50 (24.6)	< 0.001**	2.60 (1.60–4.24)
**Clinical outcome data**						
Pulmonary Embolism		61 (12.4)	15 (5.2)	46 (22.5)	< 0.001**	5.31 (2.87–9.83)
Heart Failure		63 (12.9)	25 (8.7)	38 (18.7)	0.001**	2.41 (1.41–4.15)
Acute Coronary Syndrome		89 (18.2)	26 (9.1)	59 (29.1)	< 0.001**	4.11 (2.48–6.81)
Acute Respiratory Distress Syndrome		98 (20)	15 (5.2)	83 (40.9)	< 0.001**	12.54 (6.95–22.63)
Septic Shock		36 (7.3)	3 (1.0)	33 (16.3)	< 0.001**	18.38 (5.55–60.83)
Respiratory Failure		75 (15.3)	20 (7.0)	55 (27.1)	< 0.001**	4.96 (2.86–8.60)
Myocarditis		18 (3.7)	3 (1.0)	15 (7.4)	< 0.001**	7.55 (2.16–26.45)
Acute Kidney Injury		55 (11.2)	14 (4.9)	41 (20.2)	< 0.001**	4.94 (2.61–9.33)
Death		11 (2.2)	0 (0.0)	11 (5.4)	< 0.001**	0.40 (0.36–0.45)
**Laboratory investigations:**						
^†^D-dimer (mg/l)		1.6 (1.2- 2.9)	1.5 (1.2- 2.2)	2.1 (1.3- 6.1)	< 0.001**	1.13 (1.07- 1.19)
^†^CRP (mg/l)		2.2 (1.3- 25.0)	2.1 (1.2- 8.7)	3.2 (1.6- 38.0)	< 0.001**	1.00 (1.00- 1.01)
^†^BNP (pg/ml)		64.5 (35.3- 625.4)	65.0 (36.0- 610.0)	63.0 (32.3- 648.0)	0.71	1.00 (1.00–1.00)
^†^Cardiac troponin I (ng/ml)		0.1 (0.01- 4.0)	0.01 (0.002- 0.5)	1.1 (0.05- 7.9)	< 0.001**	1.12 (1.08- 1.17)
**Echocardiographic pictures:**						
Reduced EF		166 (33.9)	88 (30.7)	78 (38.4)	0.07	1.4 (0.97- 2.06)
Diastolic Dysfunction	Grade I	213 (43.5)	123 (42.9)	90 (44.3)	0.75	1.06 (0.74- 1.53)
	Grade II	64 (13.1)	35 (12.2)	29 (14.3)	0.50	1.2 (0.71- 2.04)
	Grade III	16 (3.3)	7 (2.4)	9 (4.4)	0.22	1.9 (0.68- 5.07)
Pericardial effusion		47 (9.6)	28 (9.8)	19 (9.4)	0.90	0.96 (0.52–1.76)
Pulmonary Hypertension		100 (20.4)	46 (16)	54 (26.6)	0.004**	1.9 (1.22–2.96)
Thrombus		10 (2)	5 (1.7)	5 (2.5)	0.60	1.42 (0.41–4.99)
Dilated Left Ventricle		68 (13.9)	37 (12.9)	31 (15.3)	0.45	1.22 (0.73–2.04)
Right Ventricular Dysfunction		51 (10.4)	23 (8.0)	28 (13.8)	0.04*	1.84 (1.02–3.29)
Valvular Abnormalities		62 (12.7)	21 (7.3)	41 (20.2)	<0.001**	3.21 (1.83–5.62)
Wall Motion Abnormalities		84 (17.1)	29 (10.1)	55 (27.1)	<0.001**	3.31 (2.02–5.41)

Data are represented as Mean ± SD, Median (25th and 75th percentiles) or frequency (percent) as appropriate. Data were analyzed by.

^‡^ Independent student *t* test.

^†^ Mann-whitney U test or Chi-square test as appropriate BNP: brain natriuretic peptide; CRP: high sensitivity c-reactive protein; EF: ejection fraction. OR; Odd Ratio, C.I; Confidence Interval.

* *p* value < 0.05 is significant.

** *p* value < 0.01 is highly significant.

### Clinical presentation and associated outcomes

Patients' clinical presentations varied. One or more of the following symptoms were reported: shortness of breath (42.4%), fever (31.6%), cough (35.3%) and 188 (38.4%) had pneumonia (Supplementary Material Table S2). In-hospital mortality was 11 (2.2%) among ICU-admitted patients. Patient outcomes were reported as follows: 61 (12.4%) had PE, 63 (12.9%) had heart failure (HF), 89 (18.2%) had acute coronary syndrome (ACS), 98 (20%) had ARDS, 36 (7.3%) had septic shock, 75 (15.3%) had RF, 18 (3.7%) had myocarditis, and 55 (11.2%) had AKI (Table 1).

### Laboratory results

Hemoglobin level, white blood cell (WBC) count, leukopenia, platelet count, international normalized ratio (INR), creatinine, urea, blood sugar, and liver enzymes did not differ significantly between ICU and non-ICU patients (Supplementary Material Table S2).

However, ICU patients had significantly higher D-dimer (2.1 vs. 1.5 mg/l, *p* = 0.001), hs-CRP (3.2 vs. 2.1 mg/l, *p* = 0.001), and cardiac troponin I (1.1 vs. 0.01 ng/ml, *p* = 0.001) levels compared to non-ICU patients. In contrast, BNP, and creatine kinase levels did not differ significantly between ICU and non-ICU patients (Table 1) and (Supplementary Material Table S2).

## Electrocardiographic and echocardiographic measurements

Electrocardiograms (ECGs) showed sinus rhythm in 398 (81.2%) patients, atrial fibrillation in 18 (3.7%) patients, ACS in 59 (12%) patients and, arrythmia in 15 (3.1%) patients (Supplementary Material Table S2). LVEF was preserved in 324 (66.1%) patients and reduced (<52% for men and <54% for women) in 166 (33.9%). Diastolic function was normal in 197 (40.2%) patients, while grade 1 diastolic dysfunction was detected in 213 (43.5%), grade II in 64 (13.1%), and grade III in 16 (3.3%). Increased pulmonary artery pressure was observed in 100 patients (20.4%). Other laboratory investigations are presented in Table 1.

Pre-ICU TTE findings showed significantly higher incidence RV dysfunction (28 [13.8%] vs. 23 [8.0%] patients; *p* = 0.04) and LV regional wall motion abnormalities (55 [27.1%] vs. 29 [0.1%]; *p* < 0.001] in ICU compared to non-ICU patients (Table 1).

**Figure 2:**
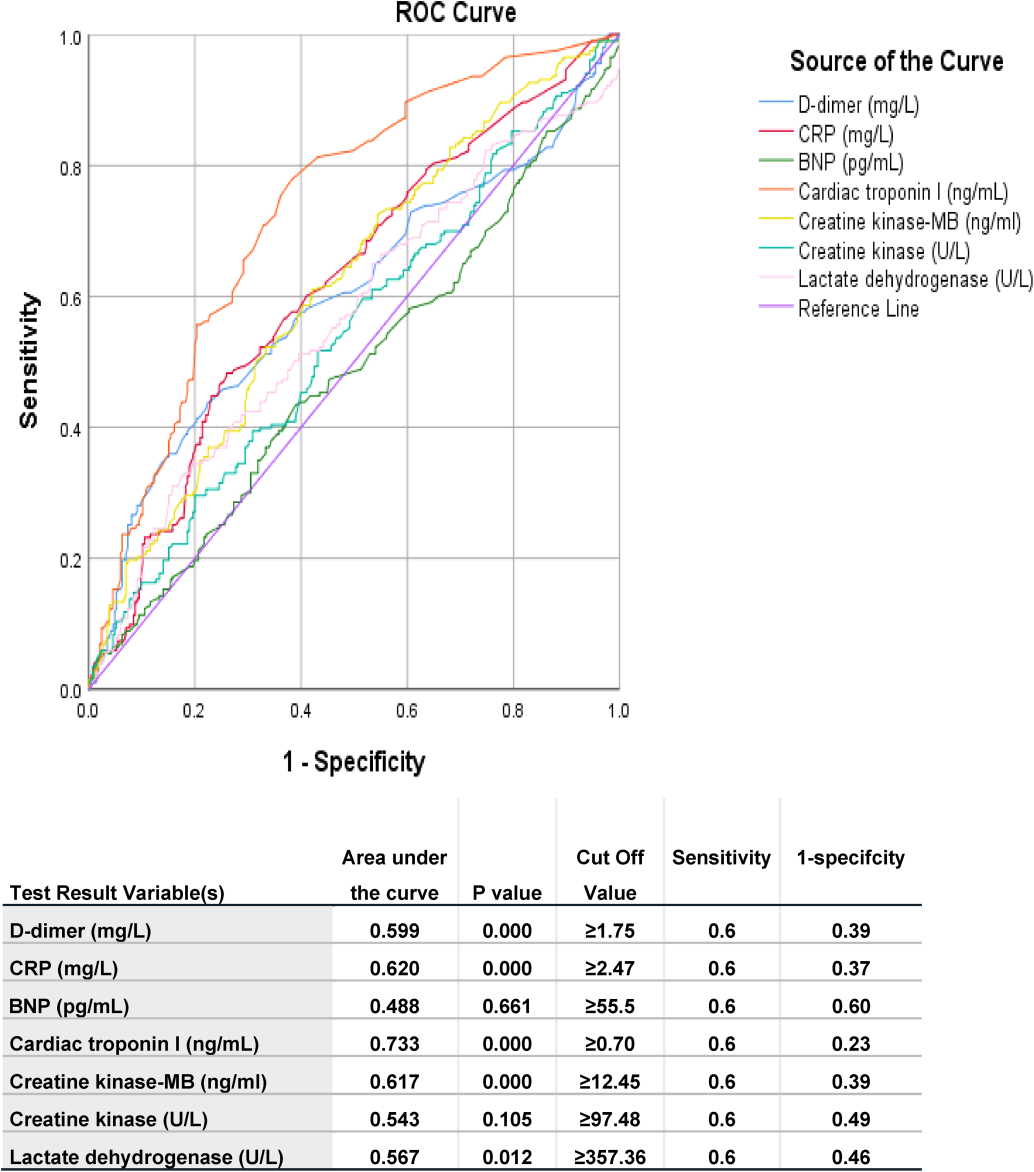
Area under ROC curve of different measured parameters of studied populations predicting ICU admission. The test result variable(s): D-dimer, CRP, BNP, Cardiac troponin I, Creatine kinase-MB, Creatine kinase, Lactate dehydrogenase has at least one tie between the positive actual state group and the negative actual state group. Statistics may be biased. BNP: brain natriuretic peptide; CRP: high sensitive c-reactive protein.

Table 2 shows predictors of mortality in the studied COVID-19 patients. Comorbidities such as chronic kidney diseases and medical history of ischemic heart disease were predictors for mortality. Moreover, clinical presentation and other factors including fever, pneumonia, PE, heart failure, acute coronary syndrome, ARDS, septic shock, elevated levels of D-dimer, CRP, BNP, pulmonary hypertension and dilated right ventricle were also predictors of mortality. Table 3 shows Association between echo-cardiographic features and inflammatory biomarkers with poor outcomes in the studied COVID-19 Patients by binary logistic regression. Echocardiographic predictors of poor outcomes were reduced LVEF (*p *= 0.037), pulmonary hypertension (*p *= 0.037), dilated right ventricle (*p *= 0.006), valvular abnormalities (*p *= 0.021), wall motion abnormalities (*p *= 0.000), D-dimer (*p *= 0.000), hs-CRP (*p *= 0.002) and BNP (*p *= 0.017) as shown in Table 3. The area under receiver operator characteristic (ROC) curves shows the most sensitive predictors of ICU admission: cardiac troponin I level (area under the curve [AUC] = 0.733, *p *= 0.000), followed by hs-CRP (AUC = 0.620, *p *= 0.000), creatine kinase-MB (AUC = 0.617, *p *= 0.000), D-dimer (AUC = 0.599, *p *= 0.000), lactate dehydrogenase (AUC = 0.567, *p *= 0.012), creatine kinase (AUC = 0.543, *p *= 0.105), and BNP (AUC = 0.488, *p *= 0.661) level as shown in Figure 2.

**Table 2:** Predictors of mortality in the studied COVID-19 patients.

Demographic data:		Hazard Ratio	
		OR (95% C.I)	*P* value
Age		0.99 (0.95- 1.03)	0.5
Sex		1.12 (0.32- 3.88)	0.9
**History of comorbidities:**			
Smoker		1.46 (0.38- 5.60)	0.6
Obesity		1.39 (0.29- 6.58)	0.7
Hypertension		0.34 (0.09- 1.28)	0.09
Chronic Kidney Disease		9.00 (2.57- 31.47)	0.001**
Diabetes Mellitus		0.38 (0.10- 1.45)	0.1
Cerebro-Vascular Accidents		2.15 (0.56- 8.32)	0.3
Ischemic Heart Disease		4.07 (1.16- 14.32)	0.02*
Lung Disease		1.90 (0.49- 7.31)	0.3
**Clinical presentation and outcome data:**			
Shortness of Breath		0.77 (0.22- 2.67)	0.7
Fever		10.26 (2.19- 48.09)	0.001**
Cough		2.24 (0.67- 7.46)	0.2
Pneumonia		4.43 (1.16- 16.91)	0.02*
Pulmonary Embolism		9.25 (2.73- 31.32)	0.001**
Heart Failure		4.07 (1.16- 14.32)	0.02*
Acute Coronary Syndrome		9.00 (2.57- 31.47)	0.001**
Acute Respiratory Distress Syndrome		11.53 (3.00- 44.31)	0.001**
Septic Shock		12.04 (3.48- 41.67)	0.001**
Respiratory Failure		2.12 (0.55- 8.18)	0.3
Myocarditis		2.72 (0.33- 22.46)	0.3
Arrhythmia		12.46 (3.38- 45.91)	0.001**
Acute Kidney Injury		7.15 (2.11- 24.28)	0.001**
**Laboratory investigations:**			
Hemoglobin, (g/dl)		0.81 (0.68- 0.98)	0.3
White Blood Cells, (10^^^9/l)		0.97 (0.82- 1.14)	0.7
Leukopenia		2.31 (0.48- 11.05)	0.3
Platelets, (10^^^9/l)		1.00 (0.99- 1.00)	0.9
INR		0.99 (0.80- 1.21)	0.9
Creatinine (mmol/l)		1.00 (1.00- 1.00)	0.7
Urea (mmol/l)		0.98 (0.95- 1.02)	0.4
Blood Sugar (mmol/l)		0.98 (0.88- 1.09)	0.7
AST (U/l)		1.00 (1.00- 1.00)	0.3
ALT (U/l)		1.00 (1.00- 1.01)	0.08
Total Bilirubin (umol/l)		1.01 (1.00- 1.01)	0.5
D-dimer (mg/l)		1.08 (1.00- 1.16)	0.05*
CRP (mg/l)		1.01 (1.00- 1.01)	0.001**
BNP (pg/ml)		1.12 (0.89- 1.78)	0.001**
Cardiac Troponin I (ng/ml)		1.05 (0.97- 1.15)	0.2
Creatine Kinase-MB (ng/ml)		1.00 (1.00- 1.01)	0.1
Creatine Kinase (U/l)		1.00 (1.00- 1.00)	0.9
Lactate Dehydrogenase (U/l)		1.00 (1.00- 1.00)	0.8
**Electrocardiographic pictures:**			
Atrial Fibrillation		0.96 (0.95- 0.98)	0.5
ACS		2.83 (0.73–10.99)	0.1
Arrhythmia		0.97 (0.95- 0.98)	0.6
**Echocardiographic pictures:**			
Reduced EF		3.52 (1.02- 12.21)	0.035
Diastolic Dysfunction	Grade I	2.32 (0.67- 8.03)	0.2
	Grade II	1.45 (0.32- 7.08)	0.6
	Grade III	0.98 (0.96–0.99)	0.5
Pericardial Effusion		0.94 (0.12- 7.52)	0.9
Pulmonary Hypertension		4.92 (1.47- 16.45)	0.004**
Thrombus		5.22 (0.60- 45.23)	0.1
Dilated Left Ventricle		2.39 (0.62- 9.24)	0.2
Dilated Right Ventricle		5.25 (1.48- 18.61)	0.004**
Vegetation		0.99 (0.98- 1.00)	0.8
Valvular Abnormalities		4.15 (1.18- 14.61)	0.02*
Wall Motion Abnormalities		4.22 (1.26- 14.17)	0.012*

Data were analyzed by Chi-square test. OR; Odd Ratio, C.I; Confidence Interval; INR: international normalized ratio; AST: Aspartate aminotransferase; ALT: Alanine aminotransferase; BNP: Brain natriuretic peptide; CRP: high sensitivity c-reactive protein; ECG: electrocardiogram; EF: ejection fraction.

* *p* value < 0.05 is significant.

** *p* value <0.01 is highly significant.

**Table 3:** Association between echo-cardiographic features and inflammatory biomarkers with poor outcomes in the studied COVID-19 patients by binary logistic regression (*N* = 263; 93 Non-ICU patients and 170 ICU patients).

Variable	B	S.E.	Wald	df	*P* value	Exp(B)	95% C.I. for EXP (B)	
							Lower	Upper
**Reduced LV Ejection Fraction**	**−0**.**403**	**0**.**194**	**4**.**335**	**1**	**0**.**037**	**0**.**668**	**0**.**457**	**0**.**977**
**Pulmonary Hypertension**	**−0**.**481**	**0**.**231**	**4**.**351**	**1**	**0**.**037**	**0**.**618**	**0**.**393**	**0**.**971**
**Dilated Right Ventricle**	**−0**.**985**	**0**.**360**	**7**.**500**	**1**	**0**.**006**	**0**.**373**	**0**.**184**	**0**.**756**
**Valvular abnormalities**	**−0**.**763**	**0**.**330**	**5**.**347**	**1**	**0**.**021**	**0**.**466**	**0**.**244**	**0**.**890**
**Wall Motion Abnormalities**	**−1**.**084**	**0**.**276**	**15**.**371**	**1**	**0**.**000**	**0**.**338**	**0**.**197**	**0**.**582**
**D-dimer (mg/l)**	**0**.**140**	**0**.**035**	**16**.**296**	**1**	**0**.**000**	**1**.**150**	**1**.**075**	**1**.**231**
**CRP (mg/l)**	**0**.**006**	**0**.**002**	**9**.**473**	**1**	**0**.**002**	**1**.**006**	**1**.**002**	**1**.**010**
**BNP (pg/mL)**	**0**.**000**	**0**.**000**	**5**.**691**	**1**	**0**.**017**	**1**.**000**	**1**.**000**	**1**.**000**

CRP, high sensitivity c-reactive protein; BNP, Brain natriuretic peptide; B, logistic regression coefficients; S.E., standard error; df, degree of freedom; Exp(B), odds ratio; C.I., confidence interval.

Dependent Variable: Poor Outcomes (including PE, HF, ARDS, septic shock, RF, myocarditis, AKI and death). *p*. value < 0.05 is significant.

The Supplementary Video shows a representative example of echocardiographic images from COVID-19 patients with poor outcomes (thrombus in the RV).

## Discussion

Our study enrolled 490 COVID-19-infected patients admitted to King Saud Medical City, of which 41.4% were admitted to the ICU. Pre-ICU TTE findings showed that patients admitted to the ICU had significantly higher incidence RV dysfunction and LV regional wall motion abnormalities than non-ICU patients. In-hospital mortality was 2.2%, with all deaths occurring in the ICU. Reduced LVEF and elevated pulmonary artery systolic pressure were found to be echocardiographic predictors of death among our studied patients.

Consistently, a recent study found that COVID-19 infection was associated with various echocardiographic abnormalities, including wall motion abnormalities, impaired LV and RV systolic and diastolic function, and pericardial effusions (22).

Our study's first key finding was the higher incidence of RV dysfunction in ICU patients, with higher pulmonary systolic pressure associated with increased mortality. This finding is consistent with Kim et al. (23), who found that 172/510 (34%) of their participants had an abnormal RV size, and Gomez et al., who reported that one-third of their participants had RV dysfunction, which was associated with 60-day mortality (odds ratio = 1.93, 95% CI: 1.13–3.3; *p* = 0.016) (24). Hypoxia caused by COVID-19 and increased pulmonary circulation demands enhanced RV afterload. The thin RV walls make it highly vulnerable to dilatation and dysfunction, with an abrupt increase in pulmonary vascular resistance and pressure secondary to hypoxia, hypercapnia, and pulmonary vascular remodeling with ARDS (24, 25).

Our study's second key finding was impaired LV systolic function in 38.4% of ICU patients. This finding is consistent with Díaz et al., who reported that LVEF was the most predictive of mortality (HR = 0.94) (26). Additionally, Jain et al. retrospectively analyzed the echocardiographic data of 72 COVID-19-positive patients, observed that about one-third had impaired LV systolic function (27). Several mechanisms have been suggested that the cause is COVD-19-related cardiac injury (28). It has been associated with increased inflammatory markers during a “cytokine storm” (29). Other studies found an association between LV systolic dysfunction and elevated inflammation biomarkers, such as CRP and troponin levels and lymphocyte percentages (CD31^+^, CD41^+^, CD81^+^, and T-cell) (30–32).

The pathogenesis of COVID-19 is thought to be connected to direct cardiac involvement *via* the angiotensin converting enzyme-2 (ACE2) signaling pathway, though the precise cause of cardiac involvement in COVID-19 is still unknown (7). The substantially elevated risk of death in this population of patients may also be explained by increased ACE2 secretion in those with cardiovascular diseases as underlying conditions (30, 33). Other theoretical explanations include cytokine-mediated harm, an imbalance between oxygen supply and demand, ischemic injury brought on by the creation of microvascular thrombi, and direct viral invasion of the myocardium (28, 34). In addition, the risk of coronary thrombotic events from atherosclerotic plaque rupture has previously been shown to be increased during viral infections (35, 36).

TTE can detect variable cardiovascular abnormalities in the clinical settings of severe COVID-19 infection. For example, TTE measurements might be crucial to distinguish underlying cardiac and non-cardiac causes in patients with RF, shock, and greatly elevated cardiac biomarkers. In addition, more abnormal echocardiogram findings were seen in patients with myocardial injury compared to those without myocardial injury. COVID-19 has a wide range of echocardiographic abnormalities, including LV dysfunction, abnormal wall motion, diastolic dysfunction, RV dysfunction, and pericardial effusions (24, 37).

Our findings showed that the inflammatory biomarkers D-dimer, hs-CRP, and BNP were associated with worse outcomes among hospitalized COVID-19 patients.

Some prognostic indicators of poor prognosis have already been identified, particularly elevated high-sensitivity troponin I levels (38). Additionally, biomarkers of worse prognosis, such as troponin I and D-dimer, are associated with RV dysfunction markers and have been proposed as independent predictors of all-cause death (39, 40). Therefore, TAPSE, which correlated with acute-phase indicators, including troponins and D-dimer (40), was the RV variable independently associated with adverse outcomes, even after adjusting for D-dimer levels (41), RV dysfunction in COVID-19 patients remained an independent predictor of death. The association between it and high-sensitivity troponin, one of the best predictors of in-hospital mortality, is supported by recent data (42). Analysis of longitudinal strain should be used to assess RV function in patients with HFrEF to enhance identifying those at risk for adverse outcomes (43). Another recent study found that right ventricular longitudinal strain (RVLS) was a strong predictor of increased mortality in patients with coronavirus illness (14, 44).

Our study highlighted the new and additive predictive significance of RV measures in patients with COVID-19 infection and underlying cardiovascular disease, in addition to confirming the function of these previously described risk predictors.

This study had a few limitations, primarily the issue of referral bias and possible confounding. TTE scans were collected using criteria implemented at our hospital during the COVID-19 outbreak (17). To reduce unnecessary exposure to healthcare professionals, TTEs were limited to ill patients (45). While this limit the generalizability of our findings to the full spectrum of COVID-19 infections, they generally represent unvaccinated, symptomatic, and hospitalized COVID-19 patients. Another limitation was that TTEs were performed as, focused scans to acquire the most critical and clinically relevant information to guide decision-making. Therefore, additional quantitative assessment techniques, such as three-dimensional scans, were unavailable. The study design, a retrospective observational study that did not include control, was another limitation of our study. Further prospective randomized controlled studies are needed and further extension of this work is warranted.

In conclusion, our study showed the likely effect of COVID-19 infection on the heart and highlighted the various echocardiographic findings of hospitalized patients. Despite the potential risk of personnel infection during the COVID-19 pandemic, TTE could be a valuable tool for initial assessment, risk stratification, and guiding patient management. Moreover, we found that lower LVEF, Dilated RV and higher pulmonary systolic pressure were echocardiographic predictor of poor outcomes that could be used as imaging markers along with elevated D-dimer, hs-CRP, and BNP levels for predicting worse outcomes and ICU admission among hospitalized COVID-19 patients.

## Data Availability

The raw data supporting the conclusions of this article will be made available by the authors, without undue reservation.
