# Supplementary material for: Association between echocardiographic features and inflammatory biomarkers with clinical outcomes in COVID-19 patients in Saudi Arabia
Source: Front Cardiovasc Med. 2023 May 26;10:1134601. doi: 10.3389/fcvm.2023.1134601 (PMC10250739; doi:10.3389/fcvm.2023.1134601)
Supplement: Supplementary file 1 [file Table1.pdf]

Supplementary Data:

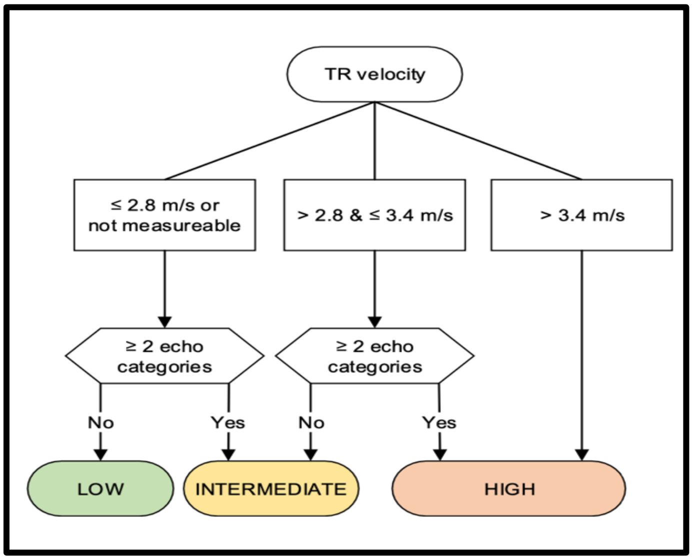

Figure S1.Supp. Flow chart to assess the probability of pulmonary hypertension using parameters identified from within ≥2 categories (the ventricles, pulmonary artery or the inferior vena cava and right atrium) in conjunction with tricuspid regurgitation velocity. Adapted from ESC/ERS Guidelines for the diagnosis and treatment of pulmonary hypertension 2015 (1,2).

Table S1.Supp. Echocardiographic signs used to help grade the probability of PH (2).

A: The ventricles[a]	B: Pulmonary artery[a]	C: Inferior vena cava and right atrium[a]
Right ventricle/left ventricle basal diameter ratio >1.0	Right ventricular outflow Doppler acceleration time <105 ms and/or mid systolic notching	Inferior vena cava diameter >21 mm with decreased inspiratory collapse (<50% with a sniff or <20% with quiet respiration)
Flattening of the interventricular septum (left ventricular eccentricity index >1.1 in systole or both systole and diastole)	Early diastolic pulmonary regurgitation (PR) velocity >2.2 m/s	Right atrial area (end systole) >18 cm²
	PA diameter >25 mm	

[a] Echocardiographic parameters from at least two different categories (A/B/C) from the list should be present to alter the level of echocardiographic probability of pulmonary hypertension.

1. Galie N, Humbert M, Vachiery JL, Gibbs S, Lang I, Torbicki A, Simonneau G, Peacock A, Vonk Noordegraaf A, Beghetti M, et al. 2015 ESC/ERS Guidelines for the diagnosis and treatment of pulmonary hypertension: the joint task force for the diagnosis and treatment of pulmonary hypertension of the European Society of Cardiology (ESC) and the European Respiratory Society (ERS): endorsed by: Association for European Paediatric and Congenital Cardiology (AEPC), International Society for Heart and Lung Transplantation (ISHLT). European Heart Journal 2016 37 67–119. (https://doi.org/10.1093/eurheartj/ehv317).
2. Augustine DX, Coates-Bradshaw LD, Willis J, Harkness A, Ring L, Grapsa J, Coghlan G, Kaye N, Oxborough D, Robinson S, Sandoval J, Rana BS, Siva A, Nihoyannopoulos P, Howard LS, Fox K, Bhattacharyya S, Sharma V, Steeds RP, Mathew T. Echocardiographic assessment of pulmonary hypertension: a guideline protocol from the British Society of Echocardiography. Echo Res Pract. 2018;5(3):G11-G24. doi: 10.1530/ERP-17-0071. PMID: 30012832; PMCID: PMC6055509.

Table S2 Supplementary: Patients' Demographics and clinical data outcomes with the risk assessment for ICU admission

Demographic data:		Descriptive			Risk assessment
	Total N=490	Non-ICU N= 287	ICU N= 203	P. value	OR (95% C.I)
**Patients' clinical presentations:**					
Shortness of Breath	208 (42.4%)	72 (25.1%)	136 (67.0%)	< 0.001**	6.06 (4.08 - 9.01)
Fever	155 (31.6%)	47 (16.4%)	108 (53.2%)	< 0.001**	5.81 (3.83 - 8.81)
Cough	173 (35.3%)	58 (20.2%)	115 (56.7%)	< 0.001**	5.16 (3.46 - 7.70)
Pneumonia	188 (38.4%)	64 (22.3%)	124 (61.1%)	< 0.001**	5.47 (3.68 - 8.13)
Arrhythmia	25 (5.1%)	4 (1.4%)	21 (10.3%)	< 0.001**	8.16 (2.76 - 24.17)
**Laboratory investigations:**					
‡ Hemoglobin, (g/dl)	11.26±6.7	11.13±2.95	11.45±9.77	0.65	0.98 (0.94- 1.03)
‡ White Blood Cells, (10^9/L)	8.92± 3.8	9.04±3.61	8.77±4.04	0.44	1.01 (0.97- 1.06)
leukopenia	44 (9%)	22 (7.7%)	22 (10.8%)	0.23	1.46 (0.79 - 2.72)
‡ Platelets, (10^9/L)	273.40±125.0	285.33±125.69	256.54±122.41	0.012	1.00 (1.00- 1.00)
† INR	1.1 (1.0 - 1.3)	1.1 (1.0- 1.3)	1.1 (1.0- 1.3)	0.21	0.85 (0.62- 1.15)
† Creatinine (mmol/L)	94.9 (68.0-191.6)	95.0 (68.0-205.8)	94.1 (68.0-162.5)	0.58	1.00 (1.00- 1.00)
† Urea (mmol/L)	9.0 (4.9-20.4)	9.0 (5.0- 30.0)	8.0 (4.8- 16.7)	0.015	0.98 (0.97- 0.99)
† Blood sugar (mmol/L)	7.6 (5.6-11.0)	7.1 (5.5- 10.3)	8.5 (5.7- 12.6)	0.006*	1.03 (1.00- 1.07)
† AST (U/L)	29.8 (18.3-56.5)	27.1 (17.6-50.2)	36.1 (20.0- 68.1)	0.001**	1.14 (0.78- 1.45)
† ALT (U/L)	25.0 (21.0-58.0)	25.0 (21.0-39.0)	26.0 (21.1- 66.0)	0.003*	1.01 (1.00- 1.01)
† Total bilirubin (umol/L)	9.8 (6.4-15.9)	9.4 (5.8- 16.3)	10.0 (7.0- 15.0)	0.48	1.00 (0.99- 1.00)
† Creatine kinase-MB (ng/ml)	11.0 (1.6-22.8)	9.2 (1.0- 19.0)	14.0 (3.1- 29.7)	< 0.001**	1.00 (1.00- 1.01)
† Creatine kinase (U/L)	104.0 (47.2-243.4)	96.0 (44.0-213.3)	115.0 (50.7-284.0)	0.09	1.00 (1.00- 1.00)
† Lactate dehydrogenase (U/L)	354.2 (213.0-588.5)	336.9 (207.0-527.0)	381.7 (231.0-672.0)	0.011	1.00 (1.00- 1.00)
**Electrocardiographic pictures:**					
Sinus rhythm	398 (81.2%)	252 (87.8%)	146 (71.9%)	< 0.001**	0.36 (0.22- 0.57)
Atrial fibrillation	18 (3.7%)	13 (4.5%)	5 (2.5%)	0.23	0.53 (0.19- 1.52)
ACS	59 (12.04%0	16 (5.6 %)	43 (21.2%)	< 0.001**	4.55 (2.48-8.35)
Arrhythmia	15 (3.1%)	6 (2.1%)	9 (4.4%)	0.14	2.17 (0.76-6.20)
**Echocardiographic pictures:**					
Vegetation	4 (0.8%)	1 (0.3%)	3 (1.5%)	0.17	4.29 (0.44 - 41.54)

Data are represented as Mean ± SD, Median (25th and 75th percentiles) or frequency (percent) as appropriate. Data were analyzed by ‡ Independent student t test, † Mann-whitney U test or Chi-square test as appropriate. INR: international normalized ratio; AST: Aspartate aminotransferase; ALT: Alanine aminotransferase. OR; Odd Ratio, C.I; Confidence Interval. * p. value <0.05 is significant, ** p. value <0.01 is highly significant.
