# Supplementary figures and images for: Association between echocardiographic features and inflammatory biomarkers with clinical outcomes in COVID-19 patients in Saudi Arabia
Source: Front Cardiovasc Med. 2023 May 26;10:1134601. doi: 10.3389/fcvm.2023.1134601 (PMC10250739; doi:10.3389/fcvm.2023.1134601)

## Slide 1
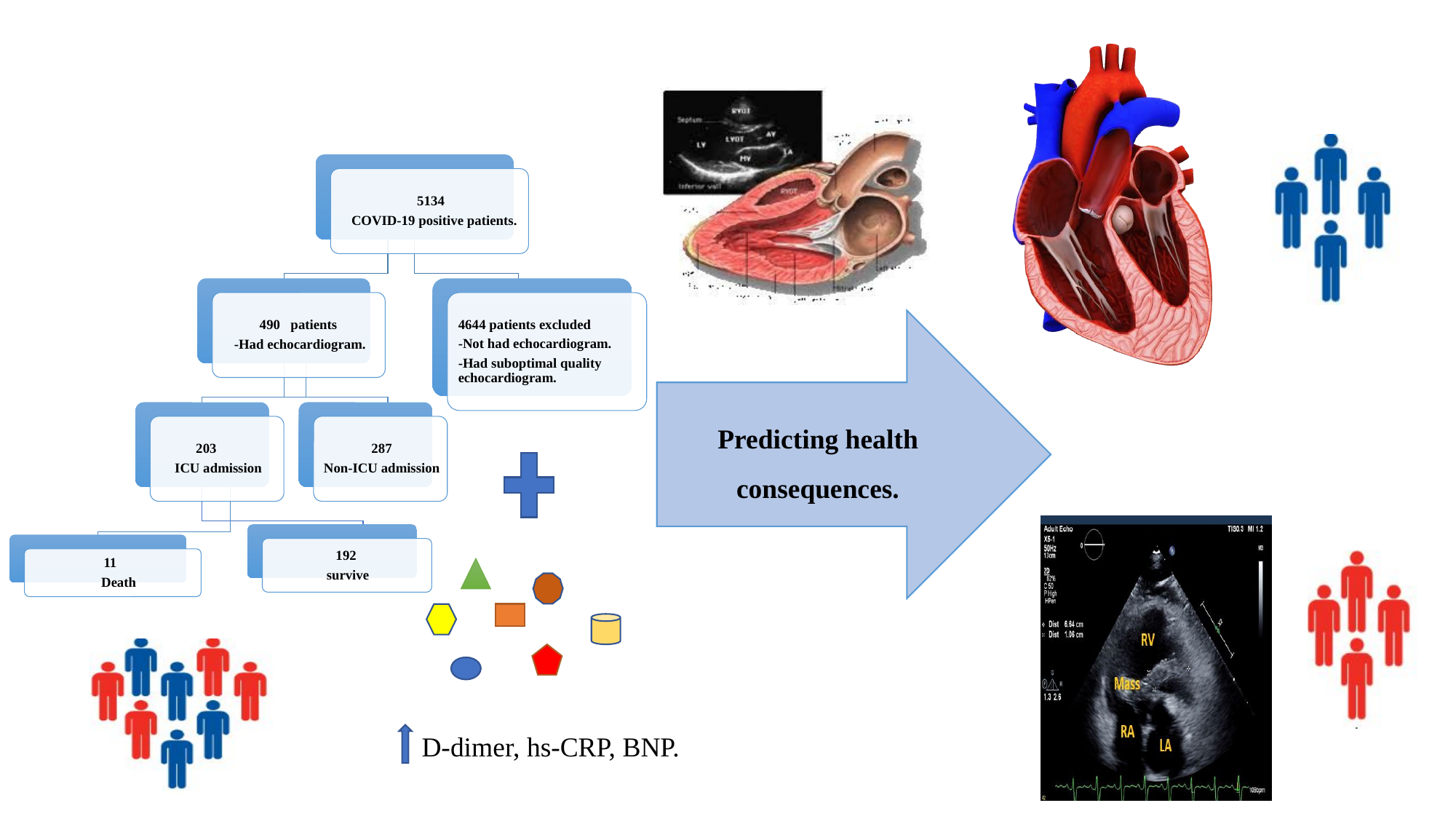

Predicting health consequences.
D-dimer, hs-CRP, BNP.

Supplement: Supplementary file 4 [file Presentation1.pptx]

## Slide 1
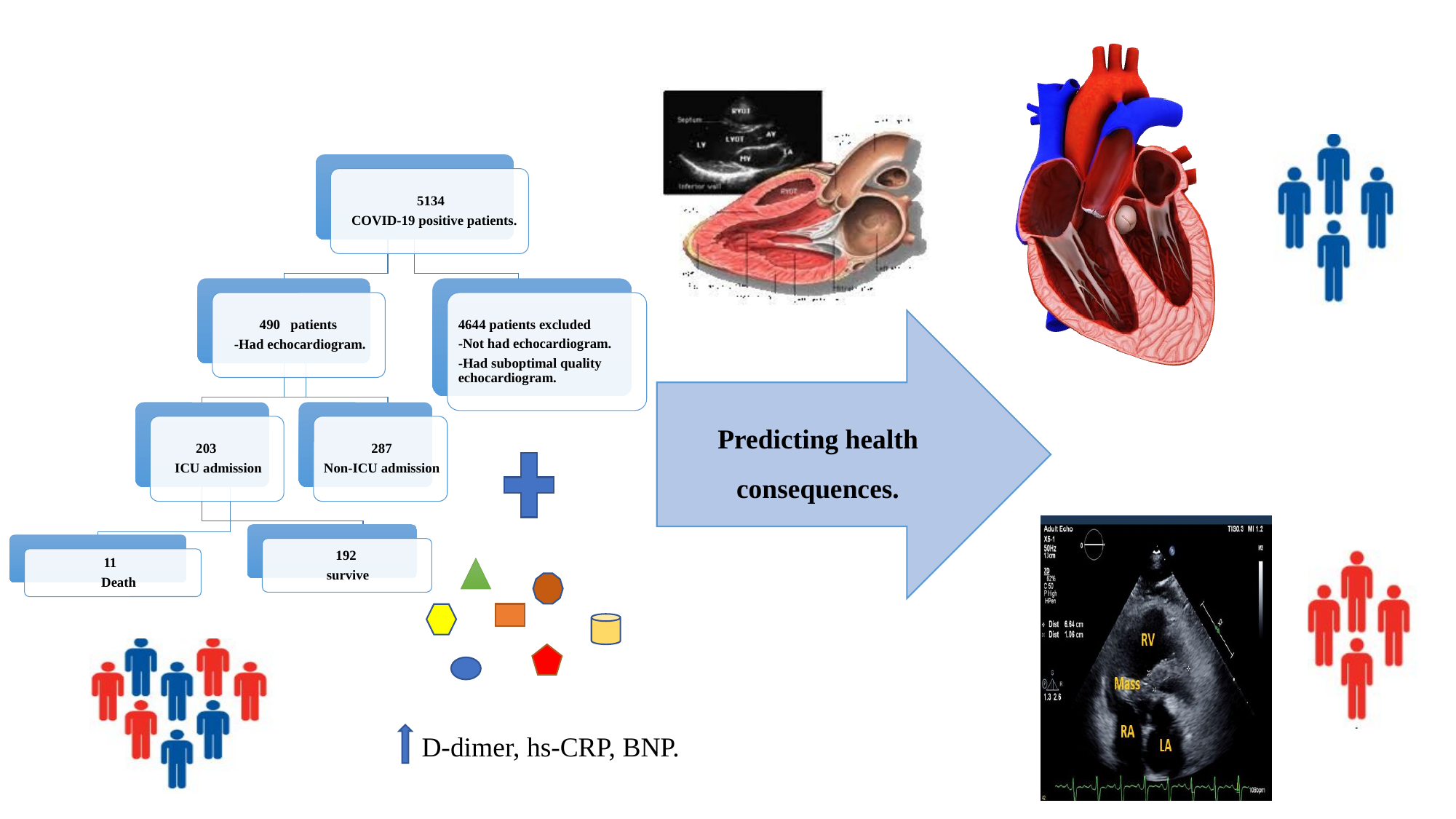

Predicting health consequences.
D-dimer, hs-CRP, BNP.

Supplement: Supplementary file 5 [file Presentation2.pptx]
